# Predictors of activities of daily living outcomes after upper limb robot-assisted therapy in subacute stroke patients

**DOI:** 10.1371/journal.pone.0193235

**Published:** 2018-02-21

**Authors:** Marco Franceschini, Michela Goffredo, Sanaz Pournajaf, Stefano Paravati, Maurizio Agosti, Francesco De Pisi, Daniele Galafate, Federico Posteraro

**Affiliations:** 1 Department of Neurorehabilitation IRCCS San Raffaele Pisana, Rome, Italy; 2 San Raffaele University, Rome, Italy; 3 Rehabilitation Medicine Service, Rehabilitation Geriatrics Department of the NHS-University Hospital of Parma, Parma, Italy; 4 Department of Neurorehabilitation Health Area of West of Tuscany, Viareggio, Italy; Harvard Medical School, UNITED STATES

## Abstract

**Background:**

Upper limb recovery is one of the main goals of post-stroke rehabilitation due to its importance for autonomy in Activities of Daily Living (ADL). Although the efficacy of upper limb Robot-assisted Therapy (RT) is well established in literature, the impact of the initial status of the patient on the effects of RT is still understudied. This paper aims to identify whether demographic, clinical and motor characteristics of stroke patients may influence the ability to independently perform ADL after RT.

**Methods:**

A retrospective study was conducted on sixty stroke patients who conducted planar upper limb goal-directed tasks with the InMotion 2.0 robot. The RT was administered 5 days/week for 4 weeks and each session lasted 45 minutes. The primary outcome measure was the Modified Barthel Index (BI), dichotomized into favourable (BI ≥75) and unfavourable (BI<75) outcomes. The potential predictors were the demographic and clinical records, and the following clinical assessment scores: Modified Ashworth Scale-Shoulder (MAS-S); Modified Ashworth Scale-Elbow (MAS-E); Fugl-Meyer Assessment Upper Extremity (FMA-UE); upper limb section of the Motricity Index (MIul); total passive Range Of Motion (pROM); and Box and Block Test (BBT).

**Results:**

Statistical analysis showed that the BBT, FMA-UE and MIul scores were significant predictors of a favourable outcome in ADL. The cut-off scores of the independent variables were calculated (FMA-UE = 32; MIul = 48; BBT = 3) with respect to the dichotomic BI outcome. Their robustness was assessed with the Fragility Index (FMA-UE = 2; MIul = 3; BBT = 7), showing that BBT is the most robust predictor of favourable BI outcome. Moreover, subjects with all predictors higher than the cut-off scores had higher probability to increase their independence in ADL at the end of the therapy. Demographic records, spasticity and pROM were not identified as predictors.

**Conclusion:**

Stroke patients with greater manual dexterity and less impairment appear to have a higher probability of achieving clinically significant ADL outcomes after upper limb RT. The obtained results can help to optimise the management of RT treatment planning. Further studies on a larger number of patients with a long-term follow up are recommended in order to evaluate other potential predictors and to validate the results.

## Introduction

Although declining stroke incidence and mortality are well documented, recent reports raised concerns that stroke incidence may be levelling off or increasing among younger adults [1,2,3,4]. Importantly, loss of arm function occurs in up to 85% of stroke survivors [5], with a significant long-term impact on Activities of Daily Living (ADL), leisure activities, and work [6]. Literature shows that only a small portion of stroke patients with upper limb motor impairment (12%) is able to regain full function, while the majority requires constant care from family or social services [7].

In order to autonomously perform ADL, the optimal restoration of arm and hand motor functions is extremely important. For this reason, many recent studies on innovative rehabilitation approaches focused on improving ADL and minimizing the hospital stay [8]. In this context, Robot-assisted Therapy (RT) could provide high-intensive, repetitive, task-specific, and interactive treatment of the impaired upper limb. Furthermore, RT is a solid, reproducible, and customizable practice for promoting motor learning [9,10]. Several RCTs and systematic reviews showed the effectiveness of upper limb RT, associated with conventional therapy, in improving the motor and functional outcomes in stroke patients, comparing it with conventional treatments [11,12,13,14]. However, despite the prominent role of RT in stroke rehabilitation, a limited number of studies aimed to identify which patient may benefit most from these innovative treatments [15,16,17,18,19]. A systematic review [16] on predictors of upper limb recovery after traditional arm therapy analysed several studies on this topic, and affirmed that measures of upper limb impairment and function were the most significant predictors (less impairment is associated with better recovery). However, literature on predictors of upper limb recovery after RT is limited and controversial [20,21,22,23,24].

Krebs *et al*. [25] analysed 208 stroke patients in order to assess if kinematic and kinetic data, automatically measured by InMotion 2.0 robot, could predict the clinical scales at the end of the RT. The results highlighted that robotic measures were able to predict the clinical measures and the recovery of patient’s motor functions.

Hisieh *et al*. [26] investigated the predictors of minimal clinically important changes on outcome measures after RT. 55 stroke patients received 20 sessions of upper limb RT with the Bi-Manu-Track (Reha-Stim, Berlin, DE) and the main outcome measures were the Fugl-Meyer Assessment Upper Extremity (FMA-UE), and the Motor Activity Log (MAL). The potential predictors were age, sex, side of lesion, time since stroke onset, finger extension, Box and Block Test (BBT) score, and FMA-UE distal score. The results revealed that greater manual dexterity (from BBT) increased the probability of achieving clinically significant motor functional improvements after RT. Furthermore, being a female was a significant predictor of MAL. Subsequently, the same group of researchers published a study [27] assessing both clinical and kinematic measures at baseline as predictors of improved motor function (FMA-UE & Wolf Motor Function Test—WMFT) and ADL (MAL & Stroke Impact Scale—SIS) after RT. Results from 66 stroke patients who conducted upper limb RT partially confirmed the previous one [26]: the manual dexterity (from BBT) was a valuable predictor of improvements at motor function and ADL. Moreover, reduced shoulder flexion synergy (measured with motion capture) was predictive of WMFT; and MAS–distal was predictive of WMFT and SIS.

Conversely, Duret *et al*. [28] reported that severely motor impaired patients may benefit more from RT. These results were obtained from a retrospective study on 17 patients, who conducted 20 sessions of upper limb RT with InMotion 2.0 robot. The correlation analysis showed that the mean value of the number of movements per robotic session decreased together with motor impairment and disability at baseline, while no correlation was found with age, aphasia and neglect. However, the improvement of the Motricity Index (MI) score and of the number of repeated movements correlated negatively with the MI at baseline and with the number of movements performed at the end of the rehabilitation period, respectively.

Therefore, considering the heterogeneity of published results on predictors of outcome measures after upper limb RT in subacute stroke patients, further studies on this topic are needed. Moreover, the efficacy of RT should be contextualised in everyday life, as suggested by the existing literature on the importance of arm function recovery in ADL [29,30,31].

The aim of this study is to identify how the characteristics of stroke patients may influence the degree of independence in ADL after upper limb RT. The identification of predictors of ADL outcomes after RT will help to identify individuals who can benefit more from this kind of therapy, and to optimise the management of treatment planning.

## Materials and methods

### Patients and experimental setup

A retrospective study was conducted on a database of 327 inpatients who were treated with the InMotion 2.0 robot (Interactive Motion Technologies, Inc., Cambridge, MA, USA) at the IRCCS San Raffaele Pisana of Rome between January 2010 and December 2016. The upper limb RT was systematically administered as part of usual care to both moderately and severely motor impaired inpatients. All subjects underwent conventional physiotherapy sessions according to the standardised rehabilitative protocol for subacute stroke patients. Inclusion criteria for the patients selection were: age between 18 and 80 years; first event of unilateral hemiparetic stroke; subacute phase (RT started within 30±7 days poststroke); upper limb Chedoke- McMaster scores between 2 and 5; RT for 17±3 sessions. Exclusion criteria were: bilateral impairment; chronic phase (RT started after 30+7 days poststroke); RT for less than 14 sessions; RT interruption for more than 3 consecutive days; incomplete data in the database.

The patients’ data had been extracted from the electronic medical records of our health care institute’s database. The following demographic and clinical records were exported from the database: age; sex; aetiology; stroke location; time between stroke and the date on which the RT began.

The following assessments were extracted at the beginning (T1) and at the end (T2) of the therapy: Modified Ashworth Scale at Shoulder (MAS-S) and Elbow (MAS-E); Modified Barthel Index (BI); Fugl-Meyer Assessment Upper Extremity (FMA-UE); Motricity Index upper limb (MIul), which assesses limbs motor impairment, and ranges from 0 to 100 per limb [32]; total passive Range Of Motion (pROM), as sum of shoulder and elbow movements (shoulder flexion/extension, abduction, intra/extra rotation and elbow extension); and Box and Block Test (BBT). All these clinical indexes were applied as routine clinical practice in our health care institute and all of them were considered as potential predictors of autonomy in ADL after RT.

The BI was defined as the primary outcome. The BI is a measure of ADL, which shows the degree of independence of a patient from any assistance.

Patients’ anonymity was preserved by identifying each record in the database with an alphanumeric code.

### Ethical considerations

Since March 2012, the Italian Data Protection Authority (Garante per la protezione dei dati personali) declared that IRCCS (Istituto di Ricovero e Cura a Carattere Scientifico—Institute for scientific research and health care) can perform retrospectives studies without the approval of the local Ethical Committee [33] since only a formal communication is needed (S2 and S3 Files). Such communication has been registered by the Ethical Committee of the IRCCS San Raffaele Pisana of Rome (date: 22/02/2017; code number: 06/17) that waived the need for participant consent.

### Rehabilitative protocol

Between T1 and T2, all selected patients performed 20 sessions (5 days/week for 4 weeks) of goal-directed, planar reaching tasks involving shoulder and elbow movements. The upper limb conducted robot-assisted movements from a central target to each of the 8 peripheral targets, equally spaced on a 0.14 m radius circumference, and viceversa, using the InMotion 2.0 robot (Interactive Motion Technologies, Inc., Cambridge, MA, USA). During each session, a series of 16 unassisted clockwise repetitions to each target followed by 3 series of 320 assisted (using an adaptive control technique) clockwise repetitions were performed by patients. At the end of each assisted series, an additional series of 16 unassisted clockwise movements was performed. After 45 minutes the session was stopped.

Furthermore, all patients underwent conventional physiotherapy sessions according to the standardised rehabilitative protocol for subacute stroke patients of IRCCS San Raffaele Pisana of Rome. Treatments were provided by senior physiotherapists and included: assisted stretching, shoulder and arm exercises, and functional reaching tasks.

### Sample size and statistical analysis

The sample size calculation (power analysis by logistic regression and by using the guidelines of Lipsey & Wilson [34] and G * Power 3.1.7 software [35]), estimated that 58 subjects would provide 80% power with 5% α and an odd-ratio of 2.5.

The primary outcome (BI at T2) was dichotomized into favourable and unfavourable. The BI cut-off scores were defined as BI ≥75 for favourable outcome and as BI < 75 for unfavourable outcome, as suggested by Uyttenboogaar *et al*. (2005) for severe stroke patients [36].

Sensitivity and specificity were defined as the rate of unfavourable and favourable outcomes, correspondingly. To investigate the relationship between sensitivity and specificity, Receiver Operator Characteristic (ROC) curves were obtained by plotting sensitivity versus 1-specificity and allowed to assess the cut-off points for each independent variable. FMA-UE, MI, pROM and BBT were dichotomized in order to find the best cut-off values at T1. MAS-S and MAS-E, on the other hand, were analysed with the Analysis Of Variance (ANOVA) because their scores were composed by 6 levels only.

Age, gender, and the clinical scales (MAS-S, MAS-E, FMA-UE, MIUL, pROM, BBT) were considered as independent variables. A multivariate analysis was performed by using logistic binary regression models in order to identify multiple relations between the dichotomized BI at T2 and the independent variables. If appropriate, the individual variables were reported with their Odds Ratio, and the significance of each coefficient in the model was examined.

We used the Fragility Index (FI), as Ridegon *et*. *al*. suggests [37], to evaluate the robustness (or fragility) in statistically significant clinical trial results [38]. The FI number indicates how many patients would be required to convert a trial from being statistically significant to not significant (p ≥ 0.05). The larger the FI the better (more robust) a trial's data are [39].

The BI Absolute Functional Gain (BI_AFG_) and the BI gain through the Montebello Rehabilitation Factor Score (BI_MRFS_) [40] were calculated for each patient as follow:

$$\begin{array}{l} {\mathrm{BI}}_{\mathrm{AFG}}=BI\left(T2\right)-BI\left(T1\right)\\ {\mathrm{BI}}_{\mathrm{MRFS}}=\frac{BI\left(T2\right)-BI\left(T1\right)}{\max\left(BI\right)-BI\left(T1\right)}\cdot 100 \end{array}$$

where BI(T1) and BI(T2) are the BI scores at T1 and T2 respectively, and max(BI) is the maximum value of the BI (i.e. 100).

## Results

### Population characteristics

**Fig 1:**
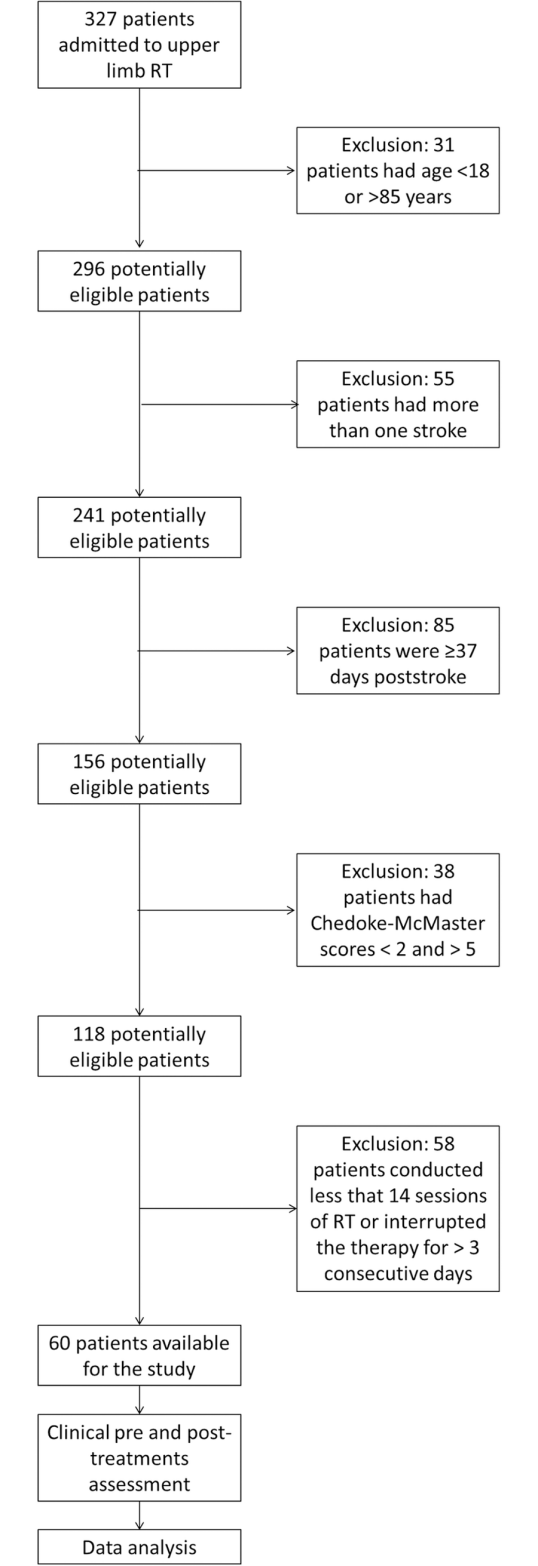
Flow chart of the step-by-step selection procedure of stroke patients included in the retrospective study.

From the 327 patients, 60 satisfied the inclusion criteria and were included in the study (S1 File, Fig 1). The mean age was 65.82 years (SD 15.40 years) and 25 (41.70%) patients were female. Half subjects were affected by stroke on the right side and half on the left one.

Table 1 shows the demographic and clinical characteristics of the sample at baseline. The BI scores at T1 are showed in Table 1: the median BI score at T1 was 24.50 with an interquartile range from 14 to 39.75.

**Table 1:** Characteristics of the sample at T1 (n = 60).

Variables	n (%)	Mean (SD)	Median (IQR)
Age, years		65.82 (15.40)	70.00 (60.00–75.00)
Gender, Male/Female	35 (58.30)/25 (41.70)		
Etiology, Ischemic/Haemorrhagic	48 (80.00)/12 (20.00)		
Lesion Side, Left/ Right	30 (50.00)/30 (50.00)		
MAS-S, 0-2/3-4	47 (78.30)/13 (21.70)		
MAS-E, 0-2/3-4	49 (81.70)/11 (18.30)		
BI		26.30 (16.50)	24.50 (14.00–39.75)
FMA-UE		28.95 (11.15)	30.00 (21.00–37.75)
MIul		47.15 (23.24)	51.00 (30.00–65.75)
pROM		781.70 (95.48)	785.00 (720.00–863.80)
BBT		6.73 (9.90)	.00 (.00–10.75)

*Abbreviation*: SD, Standard Deviation; IQR, Interquartile Range; MAS-S, Modified Ashworth Scale Shoulder; MAS-E, Modified Ashworth Scale Elbow; BI, Modified Barthel Index; FMA-UE, Fugl-Meyer Assessment Upper Extremity; MIul, Motricity Index upper limb; pROM, passive Range Of Motion; BBT, Box and Block Test.

No subject had BI ≥75 at T1, while 27 patients had BI ≥75 at T2.

### ROC curves and cut-off scores

**Fig 2:**
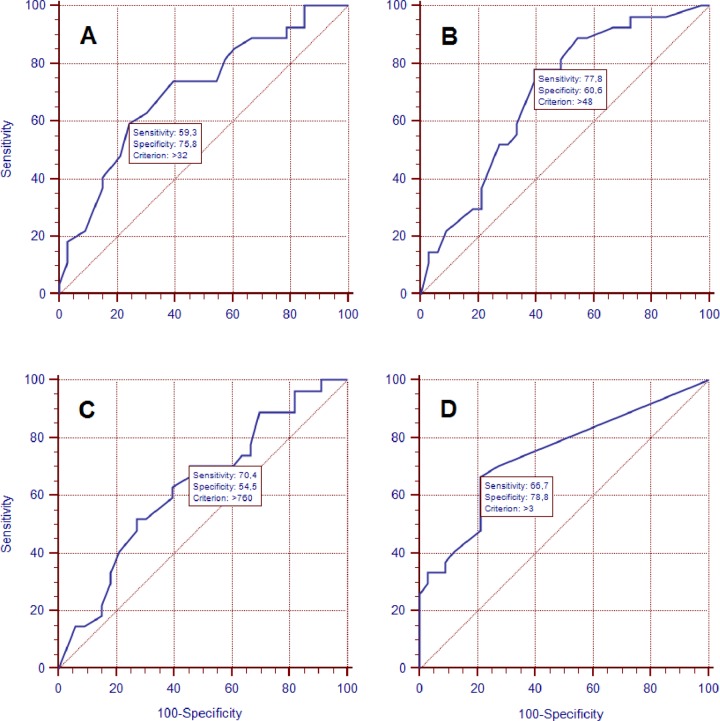
ROC curve to identify optimal criterion for variables at baseline, using characteristics of the predictive to Barthel Index at T2 ≥ 75 (A: FMA-UE; B: MIul; C: pROM; D: BBT).

The sensitivity and specificity for the cut-off scores of the independent variables (FMA-UE, MIul, BBT) in relation to the dichotomic outcome of BI (≥75 or <75) were calculated (Table 2) and plotted as ROC curves (Fig 2). The optimal cut-off score at T1 of FMA-UE was 32 (with a sensitivity of 59.3% and a specificity of 75.8%), and 27 patients were over such cut-off score; of MIul was 48 (with a sensitivity of 77.8% and a specificity of 60.5%), and 34 patients were over such cut-off score; of pROM was 760 (with a sensitivity of 70.4% and a specificity of 54.5%), and 34 patients were over such cut-off score; and of BBT was 3 (with a sensitivity of 66.7% and a specificity of 78.8%), and 25 patients were over such cut-off score.

**Table 2:** Characteristics of the predictive to Barthel Index at T2 ≥ 75 (using ROC curve optimal criterion); n = 60.

Variables	Youden Index J	Associated Criterion	# Subjects above the Criterion at T1	SE (95% CI)	SP (95%CI)	AUC (95% CI)
FMA-UE	.350	> 32	27	59.30 (38.80–77.60)	75.80 (57.70–88.90)	.704 (.572 - .815)
MIul	.384	> 48	34	77.80 (57.70–91.40)	60.60 (42.10–77.10)	.698 (.565 - .809)
pROM	.249	> 760	34	70.40 (49.80–86.20)	54.60 (36.40–71.90)	.633 (.499 - .754)
BBT	.455	> 3	25	66.70 (46.00–83.50)	78.80 (61.10–91.00)	.742 (.613 - .846)

*Abbreviation*: *CI*, confidence interval; *SE*, sensitivity; *SP*, specificity; ROC, Receiver Operating Characteristic; *AUC*, Area Under the ROC curve (maximum = 1.0); FMA-UE, Fugl-Meyer Assessment Upper Extremity; MIul, Motricity Index upper limb; pROM, passive Range Of Motion; BBT, Box and Block Test.

**Fig 3:**
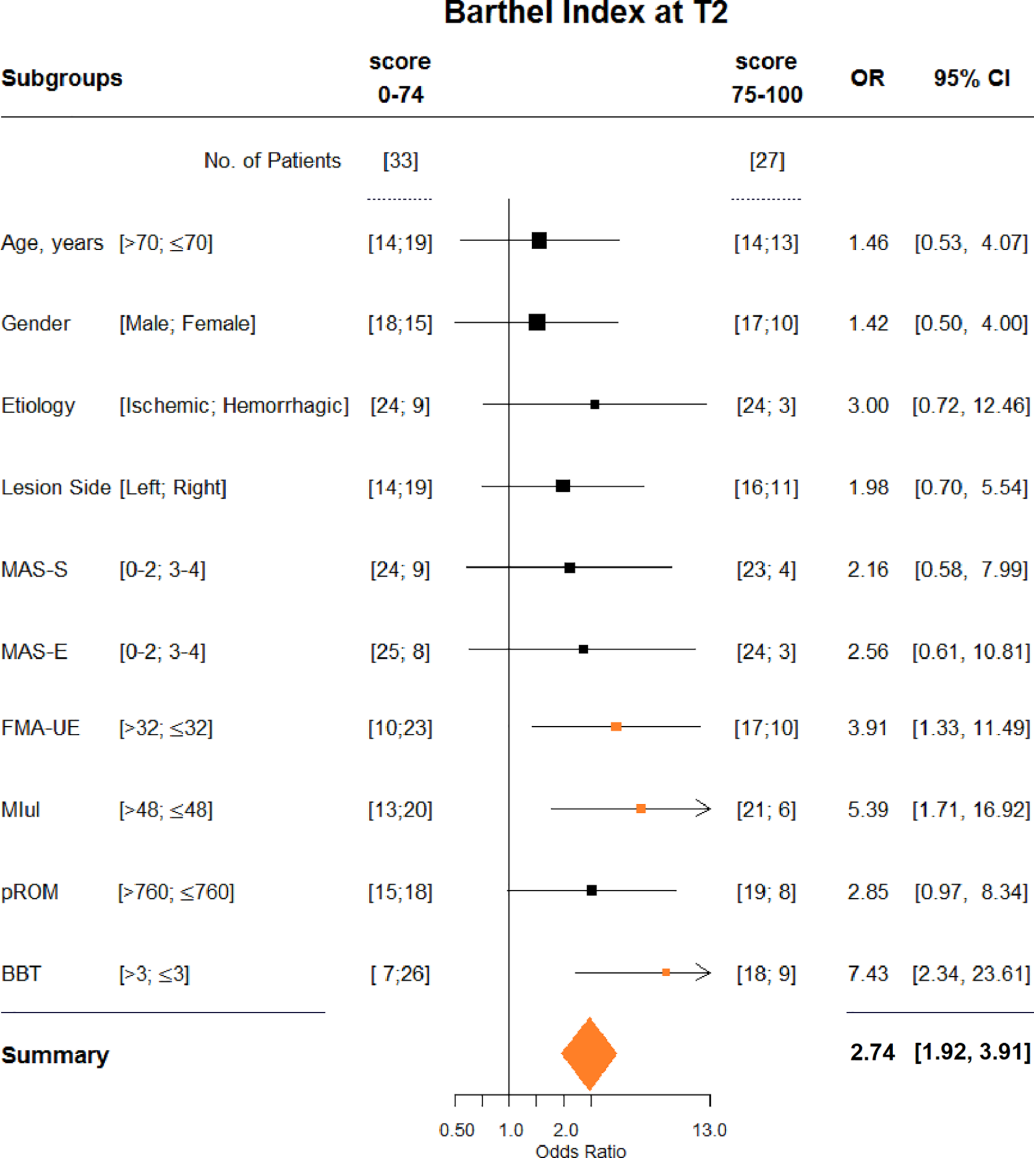
Forest-plot for predictor of independence in ADL (Barthel Index at T2 ≥ 75) for dichotomic variables; univariate analysis.

Fig 3 shows the logistic regression and depicts that FMA-UE, MIul and BBT data which are higher than the cut-off scores can significantly predict the favourable outcome (i.e. BI≥75 at T2). In particular, a BBT score higher than 3, multiplies by 7 the probability to have BI≥75 at T2. Conversely, pROM, MAS-S, MAS-E, age and gender are not significant predictors of the favourable BI at T2.

In Table 3, the number of subjects in each subgroup (e.g. 17 subjects had FM-UE > 32 and BI≥75, while 10 subjects had FM-UE ≤ 32 and BI<75, etc.) and the corresponding FI (indicating the number of patients required to lose the statistical significance) are showed. These figures have been calculated for all predictors. The robustness of BBT (FI = 7) is greater than those obtained by dichotomising FM-UE (FI = 2) and MIul (FI = 3). Moreover, having FM-UE>32, MIul>48, and BBT>3 together at T1, makes their predictive impact stronger (FI = 8).

**Table 3:** Robustness for prediction of independence in ADL (Barthel Index at T2 ≥ 75) reporting Fragility Index (FI).

			BI at T2				
			(75–100)	(0–74)	OR (95% CI)	p-value	FI
**FMA-UE**					3.910 (1.331, 11.487)	0.0131	2
	> 32		17	10			
	≤ 32		10	23			
**MIul**					5.385 (1.714, 16.919)	.0039	3
	> 48		21	13			
	≤ 48		6	20			
**BBT**					7.429 (2.338, 23.607)	0.0007	7
	> 3		18	7			
	≤ 3		9	26			
**Summary**					7.808 (2.151, 28.349)	.0018	8
FMA-UE		> 32					
MIul		> 48	14	4			
BBT		> 3					
FMA-UE		≤ 32					
MIul		≤ 48	13	29			
BBT		≤ 3					

*Abbreviation*: *OR*, Odds Ratio; *CI*, confidence interval; FI, Fragility Index; FMA-UE, Fugl-Meyer Assessment Upper Extremity; MIul, Motricity Index upper limb; BBT, Box and Block Test.

Table 4 depicts the BI_AFG_ and the BI_MRFS_ by dividing the patients accordingly to their BBT score (BBT cut-off score criteria). These figures allow to understand if the gain in ADL is different between moderately and severely motor impaired subjects. Data are shown as mean, standard deviation, median value, and 25^th^ and 75^th^ percentiles and confirms that all patients increased their ability to perform ADL at the end of the therapy. However, moderately impaired patients (BBT>3) have a higher improvement of their autonomy.

**Table 4:** BI Absolute Functional Gain (BI_AFG_) and BI Montebello Rehabilitation Factor Score (BI_MRFS_) for patients with BBT score ≤ 3 and >3.

BBT score		BI_AFG_					BI_MRFS_				
	N	Mean	SD	25th perc.	Median	75th perc.	Mean	SD	25th perc.	Median	75th perc.
**≤3**	35	38.77	21.20	18.00	43.00	51.00	51.8%	25.1%	37.5%	55.7%	71.6%
**>3**	25	50.68	22.05	38.00	49.00	64.00	71.3%	27.9%	51.9%	80.7%	88.3%

*Abbreviation*: SD, Standard Deviation; BI_AFG_, BI Absolute Functional Gain; BI_MRFS_, BI Montebello Rehabilitation Factor Score.

## Discussion

This retrospective study analysed the demographic, clinical and motor characteristics of 60 subacute stroke patients at baseline comparing them with their degree of independence in ADL after upper limb RT. Patients with a limited body function (FMA-UE, MIul) and discrete conserved dexterity (BBT) are more likely to achieve a good degree of autonomy in ADL at the end of the therapy.

A BBT score (dexterity) greater than 3 appears to raise significantly the opportunity of becoming more independent after this kind of rehabilitation. Therefore, the dexterity could be considered as a potential predictor to increase the probability of regaining independence in ADL. The results on manual dexterity are in accordance with the predictors of ADL obtained by Hisieh *et al*. [26] and Huang [27] on stroke patients who used the Bi-Manu-Track. Furthermore, our findings are in line with Kwakkel *et al*. [5], who pointed out the importance of manual dexterity in upper limb motor improvement for stroke patients, highlighting that individuals who do not show functional dexterity at stroke onset seem to have poor upper limb motor function six months after stroke. Moreover, the obtained values of BI_AFG_ and BI_MRFS_ confirm that all patients increased their ability to independently perform ADL after RT. However, subjects with a BBT score at T1 higher than the cut-off value have a higher increase of BI at T2. This finding is associated with the correlation between the ability to use hands and manipulating objects, and independence in ADL. Since a good ability to perform ADL depends on both fine (e.g. grasping) and coarse (e.g. reaching) motor abilities, and since the RT with the InMotion 2.0 robot aims to recover coarse motor abilities and do not train the fine ones, subjects who had greater level of fine motor skills at baseline have a higher probability to increase autonomy in ADL [41]. Such findings confirm the results that subjects with greater manual dexterity, and moderate impairment, appear to have a higher ability to independently perform ADL after upper limb RT.

The results earned by analysing the functional outcomes (FMA-UE and MIul) can not be compared with the studies by Hisieh *et al*. [26] and Huang [27] since they considered the motor functions as primary outcomes. The predictive power of FMA-UE and MIul are in accordance with studies on constraint-induced therapy or bilateral arm training [5,18] and with the meta-analysis by Coupar *et al*. [16]. On the other hand, our findings are in disagreement with Duret *et al*. [24], who found a negative correlation between MIul at T2 and MIul at T1. The reasons of such discrepancy can be found in the different sample sizes and in the different nature of the primary outcomes (our primary outcome is ADL, Duret’s primary outcome was motor function).

We did not find any relationship between MAS and pROM, and the improvements in daily function. The initial level of spasticity and pROM does not influence the outcome. In this context, some authors affirmed that there is a low correlation between spasticity and disabilities using the MAS and BI, as in our study [42]. Other studies on pROM and MAS showed that spasticity significantly decreases in the EG (RT) rather than in the CG (no RT), demonstrating positive effects of the RT delivered in the early phase of rehabilitation in subacute stroke patients [11,43]. Moreover, the reduction of spasticity correlates with the increase of pROM. In our study, patients were recruited within 30+7 days from the acute event and observed for about 20 days: in such period of time the spasticity may not have developed completely [44], and the muscular tone could decrease (in case of flaccidity) or increase (in case of an initial spasticity). These statements support our results on the non predictive nature of MAS and pROM in ADL outcome after RT.

The FI analysis showed that the cut-off values obtained in this study are sufficiently robust and that the subjects with FM-UE, MIul and BBT higher than such cut-off values, may be more likely to improve their independence in ADL at the end of the therapy.

Limitations of our study are correlated with the retrospective nature of the research: the presence of potential confounding factors, and a limited number of subjects. In our study, the environmental influences are minimized because all subjects were inpatients and underwent additional conventional physiotherapy sessions according to the standardised rehabilitative protocol for subacute stroke patients. However, a more controlled observational prospective study on a larger sample with a long-term follow up (6/12 months from acute event) is suggested in order to confirm our results and to study the phenomenon more accurately. In addition, other potential factors that might affect RT outcomes, such as muscle activity and kinematic parameters, need further investigation.

## Conclusions

This study reports that patients with less impairment and disability could benefit more from an upper limb rehabilitation program composed of RT and traditional therapy. Conversely, gender, age, spasticity and passive range of motion do not considerably influence the effects of such therapy on autonomy in ADL.

The obtained results can help to optimise the management of RT treatment planning, giving higher priority to patients who have more segmental motor activities and dexterity. Nevertheless, our findings confirms that patients with more severe impartment can also benefit from upper limb RT for improving their ability to perform ADL.

## Supporting information

S1 File Database (n = 60). Original data.
(XLSX)

S2 File Official documentation Italian Data Protection Authority ITA. Italian version of the Data Protection Authority (01 March 2012, Official Gazette No.72 of March 26, 2012).
(PDF)

S3 File Summary Official documentation Italian Data Protection Authority ENG. English version of the Data Protection Authority (01 March 2012, Official Gazette No.72 of March 26, 2012).
(PDF)

## References

[1] Tibæk M, Dehlendorff C, Jørgensen HS, Forchhammer HB, Johnsen SP, Kammersgaard LP Increasing incidence of hospitalization for stroke and transient ischemic attack in young adults: a registry‐based study. Journal of the American Heart Association. 2016;5(5): e003158 doi: 10.1161/JAHA.115.003158 27169547 10.1161/JAHA.115.003158 PMC4889186

[2] Fang MC, Perraillon MC, Ghosh K, Cutler DM, Rosen AB Trends in stroke rates, risk, and outcomes in the United States, 1988 to 2008. The American journal of medicine. 2014;127(7): 608–615. doi: 10.1016/j.amjmed.2014.03.017 24680794 10.1016/j.amjmed.2014.03.017 PMC4125206

[3] Khellaf M, Quantin C, d'Athis P, Fassa M, Jooste V, Hervieu M, et al Age–Period–Cohort Analysis of Stroke Incidence in Dijon From 1985 to 2005. Stroke. 2010;41(12):2762–2767. doi: 10.1161/STROKEAHA.110.592147 21071719 10.1161/STROKEAHA.110.592147

[4] Béjot Y, Delpont B, Giroud M. Rising Stroke Incidence in Young Adults: More Epidemiological Evidence, More Questions to Be Answered; 2016 doi: 10.1161/JAHA.116.003661 27169549 10.1161/JAHA.116.003661 PMC4889213

[5] Kwakkel G, Kollen BJ, van der Grond J, Prevo AJ Probability of regaining dexterity in the flaccid upper limb. Stroke. 2003;34(9):2181–2186. doi: 10.1161/01.STR.0000087172.16305.CD 12907818 10.1161/01.STR.0000087172.16305.CD

[6] Aparicio HJ, Beiser A, Himali J, Satizabal C, Pase M, Romero J, et al Temporal Trends in Stroke Incidence in the Young in the Framingham Study (I2. 003). Neurology. 2016;86(16), I2–003.

[7] Nichols-Larsen DS, Clark PC, Zeringue A, Greenspan A, Blanton S. Factors influencing stroke survivors’ quality of life during subacute recovery. Stroke. 2005;36(7):1480–148. doi: 10.1161/01.STR.0000170706.13595.4f 15947263 10.1161/01.STR.0000170706.13595.4f

[8] Kutner NG, Zhang R, Butler AJ, Wolf SL, Alberts JL, Merians AS. Quality-of-Life Change Associated With Robotic-Assisted Therapy to Improve Hand Motor Function in Patients With Subacute Stroke: A Randomized Clinical Trial. Physical Therapy. 2010;493–504. doi: 10.2522/ptj.20090160 20185616 10.2522/ptj.20090160 PMC2848350

[9] Mirelman A, Bonato P, Deutsch JE. Effects of training with a robot-virtual reality system compared with a robot alone on the gait of individuals after stroke. Stroke. 2009;40(1):169–174. doi: 10.1161/STROKEAHA.108.516328 18988916 10.1161/STROKEAHA.108.516328

[10] Brewer BR, McDowell SK, Worthen-Chaudhari LC Poststroke upper extremity rehabilitation: a review of robotic systems and clinical results. Topics in stroke rehabilitation. 2007;14(6):22–44. doi: 10.1310/tsr1406-22 18174114 10.1310/tsr1406-22

[11] Sale P, Franceschini M, Mazzoleni S, Palma E, Agosti M, Posteraro F. Effects of upper limb robot-assisted therapy on motor recovery in subacute stroke patients. Journal of neuroengineering and rehabilitation. 2014;11(1):104. 24946799 10.1186/1743-0003-11-104 PMC4074149

[12] Bovolenta F, Goldoni M, Clerici P, Agosti M, Franceschini M. Robot therapy for functional recovery of the upper limbs: a pilot study on patients after stroke. Journal of rehabilitation medicine. 2009;41(12):971–975. doi: 10.2340/16501977-0402 19841826 10.2340/16501977-0402

[13] Mehrholz J, Hädrich A, Platz T, Kugler J, Pohl M. Electromechanical and robot-assisted arm training for improving generic activities of daily living, arm function, and arm muscle strength after stroke. Cochrane Database Syst Rev. 2012;6(6). 10.1002/14651858.CD006876.pub3 22696362

[14] Norouzi-Gheidari N, Archambault PS, Fung J. Effects of robot-assisted therapy on stroke rehabilitation in upper limbs: systematic review and meta-analysis of the literature. Journal of rehabilitation research and development. 2012;49(4):479 22773253 10.1682/jrrd.2010.10.0210

[15] Dobkin BH. Progressive staging of pilot studies to improve phase III trials for motor interventions. Neurorehabilitation and neural repair. 2009;23(3):197–206. doi: 10.1177/1545968309331863 19240197 10.1177/1545968309331863 PMC4099048

[16] Coupar F, Pollock A, Rowe P, Weir C, Langhorne P. Predictors of upper limb recovery after stroke: a systematic review and meta-analysis. Clinical rehabilitation. 2012;26(4):291–313. doi: 10.1177/0269215511420305 22023891 10.1177/0269215511420305

[17] Musicco M, Emberti L, Nappi G, Caltagirone C, Italian Multicenter Study on Outcomes of Rehabilitation of Neurological Patients. Early and long-term outcome of rehabilitation in stroke patients: the role of patient characteristics, time of initiation, and duration of interventions. Archives of physical medicine and rehabilitation. 2003;84(4):551–558. doi: 10.1053/apmr.2003.50084 12690594 10.1053/apmr.2003.50084

[18] Fritz SL, Light KE, Patterson TS, Behrman AL, Davis SB. Active finger extension predicts outcomes after constraint-induced movement therapy for individuals with hemiparesis after stroke. Stroke. 2005;36(6):1172–1177. doi: 10.1161/01.STR.0000165922.96430.d0 15890987 10.1161/01.STR.0000165922.96430.d0

[19] G, Kollen B. Predicting improvement in the upper paretic limb after stroke: a longitudinal prospective study. Restorative neurology and neuroscience. 2007;25(5,6):453–460. 18334763

[20] Morone G, Bragoni M, Iosa M, De Angelis D, Venturiero V, Coiro P, et al Who may benefit from robotic-assisted gait training? A randomized clinical trial in patients with subacute stroke. Neurorehabilitation and neural repair. 2011;25(7):636–644. doi: 10.1177/1545968311401034 21444654 10.1177/1545968311401034

[21] Krebs HI, Krams M, Agrafiotis DK, DiBernardo A, Chavez JC, Littman GS, et al Robotic measurement of arm movements after stroke establishes biomarkers of motor recovery. Stroke. 2014;45(1):200–204. doi: 10.1161/STROKEAHA.113.002296 24335224 10.1161/STROKEAHA.113.002296 PMC4689592

[22] Hsieh YW, Lin KC, Wu CY, Lien HY, Chen JL, Chen CC, Chang WH. Predicting clinically significant changes in motor and functional outcomes after robot-assisted stroke rehabilitation. Archives of physical medicine and rehabilitation. 2014;95(2):316–321. doi: 10.1016/j.apmr.2013.09.018 24113336 10.1016/j.apmr.2013.09.018

[23] Huang PC, Hsieh YW, Wang CM, Wu CY, Huang SC, Lin KC. Predictors of motor, daily function, and quality-of-life improvements after upper-extremity robot-assisted rehabilitation in stroke. American Journal of Occupational Therapy. 2014;68(3):325–333. doi: 10.5014/ajot.2014.010546 24797196 10.5014/ajot.2014.010546

[24] Duret C, Hutin E, Lehenaff L, Gracies JM. Do all sub acute stroke patients benefit from robot-assisted therapy? A retrospective study. Restorative neurology and neuroscience. 2015;33(1): 57–65. doi: 10.3233/RNN-140418 25420902 10.3233/RNN-140418

[25] Krebs HI, Krams M, Agrafiotis DK, DiBernardo A, Chavez JC, Littman GS, et al Robotic measurement of arm movements after stroke establishes biomarkers of motor recovery. Stroke. 2014;45(1):200–204. doi: 10.1161/STROKEAHA.113.002296 24335224 10.1161/STROKEAHA.113.002296 PMC4689592

[26] Hsieh YW, Lin KC, Wu CY, Lien HY, Chen JL, Chen CC, Chang WH. Predicting clinically significant changes in motor and functional outcomes after robot-assisted stroke rehabilitation. Archives of physical medicine and rehabilitation. 2014;95(2):316–321. doi: 10.1016/j.apmr.2013.09.018 24113336 10.1016/j.apmr.2013.09.018

[27] Huang PC, Hsieh YW, Wang CM, Wu CY, Huang SC, Lin KC. Predictors of motor, daily function, and quality-of-life improvements after upper-extremity robot-assisted rehabilitation in stroke. American Journal of Occupational Therapy. 2014;68(3):325–333. doi: 10.5014/ajot.2014.010546 24797196 10.5014/ajot.2014.010546

[28] Duret C, Hutin E, Lehenaff L, Gracies JM. Do all sub acute stroke patients benefit from robot-assisted therapy? A retrospective study. Restorative neurology and neuroscience. 2015;33(1): 57–65. doi: 10.3233/RNN-140418 25420902 10.3233/RNN-140418

[29] Sveen U, Bautz-Holter E, Margrethe Sodring KAREN, Bruun Wyller TORGEIR, Laake K. Association between impairments, self-care ability and social activities 1 year after stroke. Disability and rehabilitation. 1999;21(8):372–377. 10503978 10.1080/096382899297477

[30] Smania N, Gambarin M, Tinazzi M, Picelli A, Fiaschi A, Moretto G, et al Are indexes of arm recovery related to daily life autonomy in patients with stroke?. European journal of physical and rehabilitation medicine. 2009;45(3):349–354. 19396056

[31] Pedersen PM, Jørgensen HS, Nakayama H, Raaschou HO, Olsen T S. Comprehensive assessment of activities of daily living in stroke. The Copenhagen Stroke Study. Archives of physical medicine and rehabilitation. 1997;78(2):161–165. 9041897 10.1016/s0003-9993(97)90258-6

[32] Wade DT, Measuring arm impairment and disability after stroke. International disability studies. 1989;11(2):89–92. 2698395 10.3109/03790798909166398

[33] Garante per la protezione dei dati personali: Autorizzazione generale al trattamento dei dati personali effettuato per scopi di ricerca scientifica—1° marzo 2012. Gazzetta Ufficiale della Repubblica Italiana 2012. Gazzetta Ufficiale della Repubblica Italiana. 2012,72:47–52.

[34] Lipsey MW. Design Sensitivity: Statistical Power for Experimental Research. Newbury Park, CA: SAGE 1990.

[35] Faul F, Erdfelder E, Buchner A, Lang AG. G*Power Version 3.1.7 [computer software]. Uiversität Kiel, Germany 2013.

[36] Uyttenboogaart M, Stewart RE, Vroomen PC, De Keyser J, Luijckx GJ. Optimizing cutoff scores for the Barthel index and the modified Rankin scale for defining outcome in acute stroke trials. Stroke. 2005;36(9):1984–1987. doi: 10.1161/01.STR.0000177872.87960.61 16081854 10.1161/01.STR.0000177872.87960.61

[37] Ridgeon EE, Young PJ, Bellomo R, Mucchetti M, Lembo R, Landoni G. The fragility index in multicenter randomized controlled critical care trials. Critical care medicine. 2016;44(7):1278–1284. doi: 10.1097/CCM.0000000000001670 26963326 10.1097/CCM.0000000000001670

[38] Walsh M, Srinathan SK, McAuley DF, Mrkobrada M, Levine O, Ribic C, et al The statistical significance of randomized controlled trial results is frequently fragile: a case for a Fragility Index. Journal of clinical epidemiology. 2014,67(6):622–628. doi: 10.1016/j.jclinepi.2013.10.019 24508144 10.1016/j.jclinepi.2013.10.019

[39] Evaniew N, Files C, Smith C, Bhandari M, Ghert M, Walsh, et al The fragility of statistically significant findings from randomized trials in spine surgery: a systematic survey. The Spine Journal. 2015;15(10):2188–2197. doi: 10.1016/j.spinee.2015.06.004 26072464 10.1016/j.spinee.2015.06.004

[40] Drubach DA. The Montebello rehabilitation factor score. J Neural Rehabil, 1994;8:881–889.

[41] Wagner JM, Lang CE, Sahrmann SA, Edwards DF, Dromerick AW. Sensorimotor impairments and reaching performance in subjects with poststroke hemiparesis during the first few months of recovery. Physical therapy. 2007;87(6):751–765. doi: 10.2522/ptj.20060135 17442839 10.2522/ptj.20060135

[42] Sommerfeld DK, Eek EUB, Svensson AK, Holmqvist LW, von Arbin MH. Spasticity after stroke. Stroke. 2004;35(1):134–139. doi: 10.1161/01.STR.0000105386.05173.5E 14684785 10.1161/01.STR.0000105386.05173.5E

[43] Bovolenta F, Sale P, Dall'Armi V, Clerici P, Franceschini M. Robot-aided therapy for upper limbs in patients with stroke-related lesions. Brief report of a clinical experience. Journal of neuroengineering and rehabilitation. 2011;8(1):18. 21477331 10.1186/1743-0003-8-18 PMC3086823

[44] Collin C, Wade DT, Davies S, Horne V. The Barthel ADL Index: a reliability study. International disability studies, 1988;10(2):61–63. 3403500 10.3109/09638288809164103

