# Supplementary material for: Predictors of activities of daily living outcomes after upper limb robot-assisted therapy in subacute stroke patients
Source: PLoS One. 2018 Feb 21;13(2):e0193235. doi: 10.1371/journal.pone.0193235 (PMC5821374; doi:10.1371/journal.pone.0193235)
Supplement: S2 File — Italian version of the Data Protection Authority (01 March 2012, Official Gazette No.72 of March 26, 2012). (PDF) [file pone.0193235.s002.pdf]

[doc. web n. 5805552]

Autorizzazione n. 9/2016 - Autorizzazione generale al trattamento dei dati personali effettuato per scopi di ricerca scientifica - 15 dicembre 2016
(Pubblicato sulla Gazzetta Ufficiale n. 303 del 29 dicembre 2016)

Registro dei provvedimenti
n. 531 del 15 dicembre 2016

IL GARANTE PER LA PROTEZIONE DEI DATI PERSONALI

Nella riunione odierna, in presenza del dott. Antonello Soro, presidente, della dott.ssa Augusta Iannini, vice presidente, della dott.ssa Giovanna Bianchi Clerici, della prof.ssa Licia Califano, componenti e del dott. Giuseppe Busia, segretario generale;

VISTO il decreto legislativo 30 giugno 2003, n. 196, Codice in materia di protezione dei dati personali (di seguito "Codice");

VISTO l'art. 99, comma 1, del Codice in base al quale il trattamento di dati personali effettuato per scopi scientifici è considerato compatibile con i diversi scopi per i quali i dati sono stati in precedenza raccolti o trattati;

VISTO l'art. 107 del Codice ove è stabilito che, fermo restando quanto previsto dall'art. 20 e fuori dei casi di particolari indagini statistiche o di ricerca scientifica previste dalla legge, il consenso dell'interessato al trattamento di dati sensibili, quando è richiesto, può essere prestato con modalità semplificate individuate dai codici di deontologia e buona condotta per i trattamenti di dati personali a scopi statistici o scientifici di cui all'art. 106 e l'autorizzazione del Garante può essere rilasciata anche ai sensi dell'art. 40;

VISTO l'art. 110, comma 1, del Codice che consente di trattare dati personali idonei a rivelare lo stato di salute per scopi di ricerca scientifica in campo medico, biomedico o epidemiologico, anche in assenza del consenso degli interessati, quando a causa di particolari ragioni non sia possibile informarli e il programma di ricerca sia oggetto di motivato parere favorevole del competente comitato etico a livello territoriale e sia autorizzato dal Garante, anche ai sensi dell'art. 40 del Codice;

VISTA l'autorizzazione del Garante n. 2/2016 al trattamento dei dati idonei a rivelare lo stato di salute e la vita sessuale e, in particolare, il punto 1.2 che autorizza il trattamento di dati personali idonei a rivelare lo stato di salute per scopi di ricerca scientifica finalizzata alla tutela della salute dell'interessato, di terzi o della collettività in campo medico, biomedico o epidemiologico, rinviando a quanto disposto dagli artt. 106, 107 e 110 del Codice in ordine alla necessità di acquisire il consenso delle persone interessate;

VISTE le disposizioni del codice di deontologia e buona condotta per i trattamenti di dati personali per scopi statistici e scientifici (Provv. n. 2 del 16 giugno 2004, in G.U. 14 agosto 2004, n. 190, Allegato A.4 al Codice e reperibile sul sito Internet dell'Autorità, doc. web n. 1556635) applicabili anche ai trattamenti di dati effettuati per scopi di ricerca medica, biomedica o epidemiologica non connessi con attività di tutela della salute svolte da esercenti professioni sanitarie od organismi sanitari, ovvero con attività comparabili in termini di significativa ricaduta personalizzata sull'interessato (artt. 11 e 2, comma 2);

VISTO l'art. 2 del d.lg. 24 giugno 2003, n. 211 e successive modifiche e integrazioni che definisce «studi osservazionali» gli studi nei quali "i medicinali sono prescritti secondo le indicazioni dell'autorizzazione all'immissione in commercio. L'assegnazione del paziente a una determinata strategia terapeutica non è decisa in anticipo da un protocollo di sperimentazione, ma rientra nella normale pratica clinica e la decisione di prescrivere il medicinale è del tutto indipendente da quella di includere il paziente nello studio. Ai pazienti non si applica nessuna procedura supplementare di diagnosi o monitoraggio";

CONSIDERATO che un elevato numero di trattamenti di dati idonei a rivelare lo stato di salute e, ove indispensabili per il raggiungimento delle finalità della ricerca, di dati idonei a rivelare la vita sessuale e l'origine razziale ed etnica, è effettuato da diversi titolari del trattamento per la conduzione di studi finalizzati alla ricerca scientifica in campo medico, biomedico o epidemiologico, non aventi significativa ricaduta personalizzata sull'interessato, che utilizzano dati raccolti in precedenza a fini di cura degli interessati o per l'esecuzione di precedenti progetti di ricerca, ovvero ricavati da campioni biologici prelevati in precedenza per finalità di tutela della salute o per l'esecuzione di precedenti progetti di ricerca;

CONSIDERATO che gli studi in questione non mirano soltanto a valutare la sicurezza e l'efficacia dei farmaci e di dispositivi medici nella pratica clinica ma anche a verificarne l'appropriatezza prescrittiva o ad indagare le relazioni tra i fattori di rischio e la salute umana, oppure vertono su eventi sanitari di tipo diagnostico, terapeutico o preventivo, ovvero sull'utilizzazione di strutture socio-sanitarie;

CONSIDERATO che, nell'ambito di tali studi, i dati oggetto di trattamento sono registrati nella documentazione clinica (o nella documentazione inerente i precedenti studi in cui sono stati raccolti) conservata presso i centri di cura partecipanti in conformità alla legge, oppure sono ricavati da campioni biologici asportati e conservati negli archivi di anatomia patologica dei medesimi centri o prelevati nell'ambito di precedenti progetti di ricerca;

VISTI gli specifici provvedimenti di autorizzazione rilasciati dall'Autorità, ai sensi degli artt. 110, comma 1 (ultima parte) e 41 del Codice,

con i quali è stata autorizzata, anche in assenza del consenso degli interessati, la conduzione di studi, non aventi significativa ricaduta personalizzata sull'interessato, effettuati con dati raccolti in precedenza per finalità di tutela della salute degli interessati o ricavati da campioni biologici prelevati in precedenza per le stesse finalità; ciò, in quanto, a causa di comprovate particolari ragioni, è stata documentata l'impossibilità di fornire loro l'informativa sul trattamento dei dati e il relativo programma di ricerca ha ricevuto un motivato parere favorevole del comitato etico territorialmente competente;

CONSIDERATO che i trattamenti dei dati sensibili possono essere autorizzati dal Garante anche d'ufficio con provvedimenti di carattere generale, relativi a determinate categorie di titolari o di trattamenti (art. 40 del Codice);

CONSIDERATO che l'autorizzazione di carattere generale rilasciata, ai sensi dell'art. 40 del Codice, l'11 dicembre 2014, in relazione ai trattamenti di dati idonei a rivelare lo stato di salute a scopi di ricerca scientifica effettuati, anche in assenza del consenso degli interessati, per la conduzione di studi, non aventi significativa ricaduta personalizzata sull'interessato, con dati raccolti in precedenza per finalità di tutela della salute o ricavati da campioni biologici prelevati in precedenza per la stessa finalità è risultata uno strumento idoneo per prescrivere misure uniformi a garanzia degli interessati, rendendo altresì superflua la richiesta di singoli provvedimenti di autorizzazione da parte di numerosi titolari del trattamento e semplificando, in maniera significativa, gli adempimenti degli obblighi relativi ai trattamenti di dati per la conduzione di ricerche mediche, biomediche o epidemiologiche;

RITENUTO opportuno pertanto, alla luce dell'esperienza maturata, rilasciare una nuova autorizzazione in sostituzione di quella in scadenza il 31 dicembre 2016, armonizzando le prescrizioni già impartite alla luce dell'esperienza maturata;

RITENUTO opportuno che anche tale nuova autorizzazione sia provvisoria e a tempo determinato, ai sensi dell'art. 41, comma 5, del Codice e, in particolare, efficace fino al 24 maggio 2018, ovvero fino alla diversa data di applicazione del Regolamento (UE) 2016/679 (relativo alla protezione delle persone fisiche con riguardo al trattamento dei dati personali, nonché alla libera circolazione di tali dati e che abroga la direttiva 95/46/CE);

RITENUTO opportuno che anche tali nuove autorizzazioni siano provvisorie e a tempo determinato, ai sensi dell'art. 41, comma 5, del Codice, e, in particolare efficaci fino al 24 maggio 2018, tenuto conto che a decorrere dal 25 maggio 2018 sarà applicabile il Regolamento (UE) 2016/679, entrato in vigore il 24 maggio 2016, relativo alla protezione delle persone fisiche con riguardo al trattamento dei dati personali, nonché alla libera circolazione di tali dati e che abroga la direttiva 95/46/CE;

RITENUTO altresì opportuno, in conformità a quanto previsto dall'art. 110 del Codice (ultima parte), prendere in considerazione ai fini della presente autorizzazione i trattamenti per scopi di ricerca scientifica preordinati alla conduzione di studi che siano oggetto di motivato parere favorevole del comitato etico competente a livello territoriale, non essendo a tal fine sufficiente la formazione del silenzio/assenso a seguito della notifica dello studio al predetto comitato;

CONSIDERATO che, in virtù dell'art. 11 del menzionato codice deontologico per i trattamenti di dati personali per scopi statistici e scientifici, il consenso non è necessario quando, ai sensi dell'art. 110 del Codice, non è possibile informare gli interessati per «motivi etici», ovvero per «motivi metodologici», ovvero per «motivi di impossibilità organizzativa»;

CONSIDERATO che, nelle richieste di autorizzazione finora sottoposte all'esame dell'Autorità, l'impossibilità di informare gli interessati è risultata giustificata sulla base di «motivi etici» ovvero di «motivi di impossibilità organizzativa»;

RITENUTO opportuno quindi, alla luce dell'esperienza maturata, che i trattamenti di dati idonei a rivelare lo stato di salute per scopi di ricerca scientifica preordinati alla conduzione di studi in campo medico, biomedico o epidemiologico in cui l'impossibilità di informare gli interessati sia giustificata da «motivi metodologici» vengano sottoposti a specifici provvedimenti di autorizzazione del Garante;

RITENUTO che ai trattamenti oggetto della presente autorizzazione devono trovare applicazione anche le cautele contenute nell'autorizzazione generale del Garante n. 2/2016 e nel codice deontologico sopra citati, in particolare per ciò che attiene ai criteri da tenere in considerazione per non rendere identificabili gli interessati nelle fasi dello studio successive all'estrapolazione dei dati e alle regole di condotta per gli incaricati e gli eventuali responsabili del trattamento;

RILEVATO che anche l'eventuale raccolta e la conservazione di campioni biologici, così come il trattamento dei dati che ne derivano, devono essere effettuati in conformità ai principi fondamentali di protezione e di sicurezza dei dati (v. Raccomandazione del Consiglio d'Europa n. R (92) 3 relativa ai test genetici ed allo screening genetico per scopi di natura sanitaria; Documento di lavoro sui dati genetici del Gruppo Art. 29 della Dir. 95/46/CE, WP 91/2004);

CONSIDERATO che l'utilizzo di campioni biologici nell'ambito di ricerche scientifiche che comportano l'estrapolazione di dati genetici deve essere effettuato nel rispetto dei limiti e delle condizioni contenute nella specifica autorizzazione generale del Garante n. 8/2016 al trattamento dei dati genetici, rilasciata in pari data alla presente autorizzazione generale ai sensi dell'art. 90 del Codice;

VISTA altresì la Raccomandazione del Consiglio d'Europa n. R (2016) 6 che individua le condizioni e i limiti nel rispetto dei quali è consentito l'utilizzo di materiale biologico per attività di ricerca, anche appartenente a persone decedute, prelevato per uno scopo diverso da quello della sua conservazione a fini di ricerca, ivi compreso quello prelevato per l'esecuzione di un precedente progetto di ricerca; considerato che tale Raccomandazione stabilisce che il materiale biologico prelevato per scopi diversi da quelli di conservazione a fini di ricerca può essere messo a disposizione per attività di ricerca con il necessario consenso dell'interessato e che, a tal fine, dovrebbe essere fatto ogni ragionevole sforzo per contattare la persona interessata, mentre ove tale contatto non sia possibile, il materiale biologico può essere utilizzato per attività di ricerca soltanto se sono soddisfatti particolari requisiti (art. 2, e Cap. III e V);

VISTO il Codice di deontologia medica del1'8 maggio 2014 nella parte in cui prevede l'obbligo del segreto professionale da parte del medico anche con riferimento ai pazienti deceduti (art. 10);

CONSIDERATO che la presente autorizzazione prende in considerazione quelle ipotesi residuali, ammesse dal Codice (art. 110), in cui il necessario consenso degli interessati per il trattamento di dati sensibili a scopi di ricerca scientifica in campo medico, biomedico o epidemiologico non sia stato previamente raccolto dai titolari del trattamento, sussistano delle particolari e comprovate circostanze dalle quali derivi l'impossibilità di informare gli interessati e la ricerca non possa essere realizzata mediante il trattamento di dati anonimi o di dati riferiti a individui che sia possibile contattare al fine di rendere l'informativa di cui all'art. 13 del Codice;

RILEVATO che, in ottemperanza ai principi, richiamati dall'art. 2, comma 2, del Codice, di semplificazione, armonizzazione ed efficacia delle modalità di esercizio dei diritti degli interessati e di adempimento degli obblighi da parte dei titolari del trattamento, è auspicabile che l'informativa sul trattamento di dati idonei a rivelare lo stato di salute per scopi di ricerca scientifica sia fornita all'interessato contestualmente a quella sul trattamento dei dati a fini di cura, specie da parte di titolari del trattamento che perseguono finalità di ricerca scientifica unitamente ad attività di ricovero e cura;

RITENUTO che, nell'ambito di detta informativa resa all'interessato, occorre distinguere chiaramente, con riferimento all'indicazione degli elementi di cui all'art. 13 del Codice, i trattamenti effettuati per scopi di ricerca scientifica da quelli preordinati alla tutela della salute, evidenziando in particolare la volontarietà della partecipazione alla ricerca, in modo da consentire all'interessato, di manifestare consapevolmente un consenso libero e specifico rispetto ai differenti scopi perseguiti (artt. 13, 23, 78, comma 5, e 105, comma 2, del Codice);

VISTO l'art. 11, comma 2, del Codice, il quale stabilisce che i dati trattati in violazione della disciplina rilevante in materia di trattamento di dati personali non possono essere utilizzati;

VISTI gli artt. 31 e seguenti del Codice e il disciplinare tecnico di cui all'Allegato B) al medesimo Codice in materia di misure minime di sicurezza;

VISTI gli artt. 20, 26, 40, 41, 98, 107 e 110 del Codice;

VISTI gli artt. 162, comma 2-bis, 167, comma 2, e 170 del Codice che sanzionano il trattamento illecito dei dati personali e l'inosservanza dei provvedimenti del Garante;

VISTI gli atti d'ufficio;

VISTE le osservazioni dell'Ufficio formulate dal segretario generale ai sensi dell'art. 15 del regolamento del Garante n. 1/2000;

Relatore la prof.ssa Licia Califano;

AUTORIZZA

il trattamento dei dati idonei a rivelare lo stato di salute e, solo ove indispensabili per il raggiungimento delle finalità della ricerca, dei dati idonei a rivelare la vita sessuale e l'origine razziale ed etnica, anche in assenza del consenso degli interessati, per scopi di ricerca scientifica in campo medico, biomedico o epidemiologico nel rispetto dei limiti e delle condizioni di seguito indicate.

1. Ambito di applicazione.

La presente autorizzazione è rilasciata:

a) alle università, agli altri enti o istituti di ricerca e società scientifiche, nonché ai ricercatori che operano nell'ambito di dette università, enti, istituti di ricerca e ai soci di dette società scientifiche;

b) agli esercenti le professioni sanitarie e agli organismi sanitari nei limiti di cui all'art. 2, comma 2, del codice di deontologia e buona condotta per i trattamenti di dati personali per scopi statistici e scientifici (Allegato A.4 al Codice).

Il trattamento dei dati oggetto della presente autorizzazione può essere altresì effettuato da persone fisiche o giuridiche, enti, associazioni e organismi privati, nonché da soggetti specificatamente preposti al trattamento quali incaricati o responsabili (ricercatori, monitor, commissioni di esperti, organizzazioni di ricerca a contratto, laboratori di analisi, ecc.) (artt. 4, comma 1, lett. f), 28, 29 e 30 del Codice).

2. Finalità del trattamento: ricerca scientifica in campo medico, biomedico o epidemiologico.

1. La presente autorizzazione è rilasciata quando:

- il trattamento è necessario per la conduzione di studi, non aventi significativa ricaduta personalizzata sull'interessato, effettuati con dati raccolti in precedenza a fini di cura della salute o per l'esecuzione di precedenti progetti di ricerca ovvero ricavati da campioni biologici prelevati in precedenza per finalità di tutela della salute o per l'esecuzione di precedenti progetti di ricerca e

- la ricerca è effettuata sulla base di un progetto, oggetto di motivato parere favorevole del competente comitato etico a livello territoriale, secondo le modalità di cui all'art. 3 del codice di deontologia e buona condotta per i trattamenti di dati personali per scopi statistici e scientifici (Allegato A.4 al Codice).

Gli studi oggetto della presente autorizzazione possono riguardare anche le relazioni tra i fattori di rischio e la salute umana, o mirano a valutare la sicurezza e l'efficacia di farmaci o di dispositivi medici nella pratica clinica, o a verificare l'appropriatezza prescrittiva, oppure vertono su eventi sanitari di tipo diagnostico, terapeutico o preventivo, ovvero sull'utilizzazione di strutture socio-sanitarie.

2. La presente autorizzazione non riguarda gli scopi della ricerca che possono essere realizzati, nel caso concreto, mediante:

- il trattamento di dati anonimi;
- il trattamento di dati riferiti ad interessati che sia possibile contattare al fine di rendere l'informativa e acquisirne il consenso.

3. Categorie di dati oggetto di trattamento.

Prima di iniziare o proseguire il trattamento i sistemi informativi e i programmi informatici sono configurati riducendo al minimo l'utilizzazione di dati personali e di dati identificativi, in modo da escluderne il trattamento quando le finalità perseguite nei singoli casi possono essere realizzate mediante, rispettivamente, dati anonimi od opportune modalità che permettano di identificare l'interessato solo in caso di necessità, in conformità all'art. 3 del Codice.

Il trattamento può riguardare unicamente i dati personali strettamente pertinenti ai sopra indicati scopi, ivi compresi quelli ricavati da campioni biologici, salvo che questi non costituiscano «dati genetici» ai sensi dell'autorizzazione n. 8/2016 rilasciata dal Garante in virtù dell'art. 90 del Codice .

Il trattamento di dati genetici resta infatti autorizzato nei limiti e alle condizioni individuati nella predetta autorizzazione.

4. Impossibilità di informare gli interessati.

L'autorizzazione riguarda il trattamento dei dati degli interessati da includere nella ricerca che non è possibile contattare al fine di fornire l'informativa - a causa della sussistenza di una delle seguenti ragioni, considerate del tutto particolari o eccezionali, documentate nel progetto di ricerca:

1. Motivi etici riconducibili alla circostanza che l'interessato ignora la propria condizione. Rientrano in questa categoria le ricerche per le quali l'informativa sul trattamento dei dati da rendere agli interessati comporterebbe la rivelazione di notizie concernenti la conduzione dello studio la cui conoscenza potrebbe arrecare un danno materiale o psicologico agli interessati stessi (possono rientrare in questa ipotesi, ad esempio, gli studi epidemiologici sulla distribuzione di un fattore che predica o possa predire lo sviluppo di uno stato morboso per il quale non esista un trattamento).

2. Motivi di impossibilità organizzativa riconducibili alla circostanza che la mancata considerazione dei dati riferiti al numero stimato di interessati che non è possibile contattare per informarli, rispetto al numero complessivo dei soggetti che si intende coinvolgere nella ricerca, produrrebbe conseguenze significative per lo studio in termini di alterazione dei relativi risultati; ciò avuto riguardo, in particolare, ai criteri di inclusione previsti dallo studio, alle modalità di arruolamento, alla numerosità statistica del campione prescelto, nonché al periodo di tempo trascorso dal momento in cui i dati riferiti agli interessati sono stati originariamente raccolti (ad esempio, nei casi in cui lo studio riguarda interessati con patologie ad elevata incidenza di mortalità o in fase terminale della malattia o in età avanzata e in gravi condizioni di salute).

Con riferimento a tali motivi di impossibilità organizzativa, è autorizzato il trattamento dei dati di coloro i quali, all'esito di ogni ragionevole sforzo compiuto per contattarli, anche attraverso la verifica dello stato in vita, la consultazione dei dati riportati nella documentazione clinica, l'impiego dei recapiti telefonici eventualmente forniti, nonché l'acquisizione dei dati di contatto presso l'anagrafe degli assistiti o della popolazione residente, risultino essere al momento dell'arruolamento nello studio:

- deceduti o

- non contattabili.

Resta fermo l'obbligo di raccogliere il consenso al trattamento dei dati degli interessati inclusi nella ricerca in tutti i casi in cui, nel corso dello studio, sia possibile rendere loro un'adeguata informativa e, in particolare, laddove questi si rivolgano al centro di cura, anche per visite di controllo.

5. Modalità di trattamento.

Il trattamento dei dati oggetto della presente autorizzazione è effettuato nel rispetto delle disposizioni del codice di deontologia e buona condotta per i trattamenti di dati personali per scopi statistici e scientifici (Allegato A.4 al Codice) e unicamente con operazioni strettamente indispensabili alla conduzione dello studio.

Ove la ricerca non possa raggiungere i suoi scopi senza l'identificazione, anche temporanea, degli interessati, nel trattamento successivo alla raccolta retrospettiva dei dati, sono adottate tecniche di cifratura o utilizzati codici identificativi oppure altre soluzioni che, considerato il numero dei dati trattati, li rendono non direttamente riconducibili agli interessati, permettendo di identificare questi ultimi solo in caso di necessità. In questi casi, i codici utilizzati non sono desumibili dai dati personali identificativi degli interessati, salvo che ciò risulti impossibile in ragione delle particolari caratteristiche del trattamento o richieda un impiego di mezzi manifestamente sproporzionato e sia motivato, altresì, per iscritto, nel progetto di ricerca.

L'abbinamento al materiale di ricerca dei dati identificativi dell'interessato, sempre che sia temporaneo ed essenziale per il risultato della ricerca, è motivato, inoltre, per iscritto.

6. Comunicazione e diffusione.

I soggetti individuati al paragrafo 1) che agiscono in qualità di titolari del trattamento, anche unitamente ad altri titolari, possono comunicare tra loro i dati personali oggetto della presente autorizzazione nella misura in cui rivestano il ruolo di promotore, di centro coordinatore o di centro partecipante e l'operazione di comunicazione sia indispensabile per la conduzione dello studio.

I dati idonei a rivelare lo stato di salute degli interessati, nonché quelli relativi alla vita sessuale e all'origine razziale ed etnica utilizzati per la conduzione dello studio non possono essere diffusi. I risultati delle ricerche possono essere diffusi in forma aggregata, ovvero secondo modalità che non rendano identificabili gli interessati neppure tramite dati identificativi indiretti, anche nell'ambito di pubblicazioni.

7. Conservazione dei dati e dei campioni.

Nel quadro del rispetto dell'obbligo previsto dall'art. 11, comma 1, lett. e) del Codice, i dati e i campioni biologici utilizzati per l'esecuzione della ricerca sono conservati mediante tecniche di cifratura o l'utilizzazione di codici identificativi oppure di altre soluzioni che, considerato il numero dei dati e dei campioni conservati, non li rendono direttamente riconducibili agli interessati, per un periodo di tempo non superiore a quello necessario agli scopi per i quali essi sono stati raccolti o successivamente trattati.

A tal fine, è indicato nel progetto di ricerca il periodo di conservazione, successivo alla conclusione dello studio, al termine del quale i predetti dati e campioni sono trasformati in forma anonima.

8. Custodia e sicurezza.

Fermo restando l'obbligo di adottare le misure minime di sicurezza previste dal Codice (artt. 33-35 e All. B) al Codice), sono impiegati dal/i titolare/i del trattamento, ciascuno per la parte di propria competenza in relazione al ruolo ricoperto nel trattamento dei dati e alle conseguenti responsabilità, specifiche misure e accorgimenti tecnici per incrementare il livello di sicurezza dei dati trattati per l'esecuzione dello studio in conformità alle indicazioni contenute nelle "Linee guida per i trattamenti di dati personali nell'ambito delle sperimentazioni cliniche di medicinali" adottate dal Garante con Provvedimento del 24 luglio 2008 (doc. web n. 1533155).

Ciò, sia nella fase di memorizzazione o archiviazione dei dati (e, eventualmente, di raccolta e conservazione dei campioni biologici) sia nella fase successiva di elaborazione delle medesime informazioni, nonché nella successiva fase di trasmissione dei dati al promotore o ai soggetti esterni che collaborano con il primo per l'esecuzione dello studio. Sono adottati, in particolare:

a. idonei accorgimenti per garantire la protezione dei dati dello studio dai rischi di accesso abusivo ai dati, furto o smarrimento parziali o integrali dei supporti di memorizzazione o dei sistemi di elaborazione portatili o fissi (ad esempio, attraverso l'applicazione parziale o integrale di tecnologie crittografiche a file system o database, oppure tramite l'adozione di altre misure informatiche di protezione che rendano inintelligibili i dati ai soggetti non legittimati) nelle operazioni di registrazione e archiviazione dei dati effettuate mediante strumenti elettronici;

b. protocolli di comunicazione sicuri basati sull'utilizzo di standard crittografici nella trasmissione elettronica dei dati raccolti nell'ambito dello studio a un database centralizzato in cui sono memorizzati o archiviati, nonché nella trasmissione in via telematica dei dati dello studio al promotore o ai soggetti esterni di cui lo stesso promotore si avvale per la conduzione dello studio. Laddove detta trasmissione sia effettuata mediante supporto ottico (CD-ROM) è designato un incaricato della ricezione presso il promotore ed è utilizzato, per la condivisione della chiave di cifratura dei dati, un canale di trasmissione differente da quello utilizzato per la trasmissione del contenuto;

c. tecniche di etichettatura, nella conservazione e nella trasmissione di campioni biologici, mediante codici identificativi, oppure altre soluzioni che, considerato il numero di campioni utilizzati, li rendono non direttamente riconducibili agli interessati, permettendo di identificare questi ultimi solo in caso di necessità;

d. con specifico riferimento alle operazioni di elaborazione dei dati dello studio memorizzati su un database centralizzato, è necessario adottare:

- idonei sistemi di autenticazione e di autorizzazione per gli incaricati in funzione dei ruoli e delle esigenze di accesso e trattamento, avendo cura di utilizzare credenziali di validità limitata alla durata dello studio e di disattivarle al termine dello stesso;

- procedure per la verifica periodica della qualità e coerenza delle credenziali di autenticazione e dei profili di autorizzazione assegnati agli incaricati del trattamento;

- sistemi di audit log per il controllo degli accessi al database e per il rilevamento di eventuali anomalie.

9. Trasferimento all'estero.

Ove sia essenziale per il risultato della ricerca trasferire dati idonei a rivelare lo stato di salute, nonché quelli relativi alla vita sessuale e all'origine razziale ed etnica degli interessati inclusi nello studio in Paesi non appartenenti all'Unione europea, in assenza del consenso espresso, in forma scritta, degli interessati (art. 43, comma 1, lett. a), del Codice), il trasferimento è autorizzato dal Garante in presenza degli

ulteriori presupposti di cui all'art. 43 del Codice, o in conformità agli artt. 44 e 45 del Codice.

10. Richieste di autorizzazione.

I titolari del trattamento che rientrano nell'ambito di applicazione della presente autorizzazione non sono tenuti a presentare una richiesta di autorizzazione a questa Autorità, qualora il trattamento che si intende effettuare sia conforme alle sue prescrizioni.

Le richieste di autorizzazione pervenute o che perverranno, anche successivamente alla data di adozione del presente provvedimento, devono intendersi accolte nei termini di cui al provvedimento medesimo.

Il Garante non prenderà in considerazione richieste di autorizzazione, ai sensi dell'art. 110 del Codice (ultima parte), per trattamenti da effettuarsi in difformità alle prescrizioni del presente provvedimento, salvo che, ai sensi dell'art. 41 del Codice, il loro accoglimento sia giustificato da circostanze o da situazioni non considerate nella presente autorizzazione.

11. Efficacia temporale.

La presente autorizzazione ha efficacia dal 1° gennaio 2017 fino al 24 maggio 2018, tenuto conto che a decorrere dal 25 maggio 2018 sarà applicabile il Regolamento (UE) 2016/679 (relativo alla protezione delle persone fisiche con riguardo al trattamento dei dati personali, nonché alla libera circolazione di tali dati e che abroga la direttiva 95/46/CE) entrato in vigore il 24 maggio 2016, salve le modifiche che il Garante ritenga di dover apportare in conseguenza di eventuali novità normative rilevanti in materia e ferme restando le determinazioni eventualmente adottate dall'Autorità in applicazione del citato Regolamento.

La presente autorizzazione sarà pubblicata nella Gazzetta Ufficiale della Repubblica italiana.

Roma, 15 dicembre 2016

IL PRESIDENTE
Soro

IL RELATORE
Califano

IL SEGRETARIO GENERALE
Busia
