# Supplementary material for: Predictors of activities of daily living outcomes after upper limb robot-assisted therapy in subacute stroke patients
Source: PLoS One. 2018 Feb 21;13(2):e0193235. doi: 10.1371/journal.pone.0193235 (PMC5821374; doi:10.1371/journal.pone.0193235)
Supplement: S3 File — English version of the Data Protection Authority (01 March 2012, Official Gazette No.72 of March 26, 2012). (PDF) [file pone.0193235.s003.pdf]

English summary of Italian Data Protection Authority
(01 March 2012, Official Gazette No.72 of March 26, 2012)

Since March 1, 2012 it is possible to conduct retrospective observational studies - subject to certain conditions - without seeking specific consent. The Italian Data Protection Authority has published the general licence to process personal data for scientific research purposes (resolution 01 March 2012, Official Gazette No.72 of March 26, 2012, http://www.garanteprivacy.it/garante/doc.jsp?ID=1878276)

The retrospective observational studies, based on groups of individuals known as "cases" or "controls", at the beginning of the study, have the following advantages:

- providing a relatively quick result, since at the beginning of the study, the necessary time for events to occur has already elapsed;
- being relatively easy to apply also in the case of rare diseases investigations;
- giving the opportunity to evaluate the effects of external factors or the outcomes of definite therapeutic strategies on a large sample of patients and also over long periods;
- having economic features.

The data collecting without seeking specific consent is allowed only if the study meets all of the following conditions:

- to belong to one of the following structures: Universities, Research Institutes, Scientific Societies, Health care centers, Private Organizations (eg CRO);
- to have scientific purpose in medical, biomedical and epidemiological fields;
- to have obtained the favorable opinion of the relevant Ethics Committee;
- to process personal data or biological samples but not genetic data;
- to have no possibility to inform interested parties for "ethical reasons" or "organizational impossibility".

What does "ethical reasons" mean?
Where interested parties ignore their condition or whether providing any information would lead material or psychological damage. For example: epidemiological studies on the distribution of a predicting factor of a disease for which there is no effective treatment.

What does "organizational impossibility" mean?
In cases where, for example, the sample size is so wide that requiring consent to all patients would undermine the study conduction, or when investigating diseases with a high mortality rate [Proportion between the number of deceased persons and The number of subjects present in a population defined over a given period of time. It is said crude when it is calculated on the whole population.] and so it is very likely that the interested parties will no longer be able to provide an authorization. However, the requirement to obtain the consent of patients who are available remains confirmed.
Moreover, Guarantor's authorization points up the modality of treatment and the data and samples preservation as well as their safekeeping and security. Authorization, in fact, "obligates those who carry out such studies to take specific measures to ensure that the processed data and biological samples can not be directly attributed to patients (e.g. by cryptographic techniques or unique identification codes).
The data and biological samples safety must be guaranteed in every phase of study, by adopting appropriate arrangements to ensure the risks of unauthorized access, theft or loss (for example by

applying cryptographic technologies or protective measures which make them unintelligible to unauthorized personnel).
Authentication systems will be required for those who handle data and tracking procedures for databases access. Similar warnings should be used in electronic data transmission to the research sponsor or central database where they are stored and archived . "

In the end, it is stipulated that to transfer the data outside the European Union - data for which consent has not been requested to the party concerned - an explicit authorization for the Guarantor should be required.

From the world of clinical research and that of the Ethics Committees, this decision has been seen as a long-awaited step towards simplifying the data collection for retrospective investigations. Ethics Committees which did not do it routinely will have to equip themselves to evaluate henceforth also retrospective studies.
